# Expression of microRNAs and Other Small RNAs in Prefrontal Cortex in Schizophrenia, Bipolar Disorder and Depressed Subjects

**DOI:** 10.1371/journal.pone.0086469

**Published:** 2014-01-27

**Authors:** Neil R. Smalheiser, Giovanni Lugli, Hui Zhang, Hooriyah Rizavi, Edwin H. Cook, Yogesh Dwivedi

**Affiliations:** Department of Psychiatry and Psychiatric Institute, University of Illinois at Chicago, Chicago, Illinois, United States of America; National Institutes of Health, United States of America

## Abstract

Because of the role played by miRNAs in post-transcriptional regulation of an array of genes, their impact in neuropsychiatric disease pathophysiology has increasingly been evident. In the present study, we assessed microRNA expression in prefrontal cortex (Brodmann area 10) of a well-characterized cohort of major depressed, bipolar, and schizophrenia subjects (obtained from Stanley Neuropathology Consortium; n = 15 in each group), using high throughput RT-PCR plates. Discrete miRNA alterations were observed in all disorders, as well as in suicide subjects (pooled across diagnostic categories) compared to all non-suicide subjects. The changes in the schizophrenia group were partially similar to those in the bipolar group, but distinct from changes in depression and suicide. Intriguingly, those miRNAs which were down-regulated in the schizophrenia group tended to be synaptically enriched, whereas up-regulated miRNAs tended not to be. To follow this up, we purified synaptosomes from pooled samples of the schizophrenia vs. control groups and subjected them to Illumina deep sequencing. There was a significant loss of small RNA expression in schizophrenia synaptosomes only for certain sequence lengths within the miRNA range. Moreover, 73 miRNAs were significantly down-regulated whereas only one was up-regulated. Strikingly, across all expressed miRNAs in synaptosomes, there was a significant inverse correlation between the fold-change of a given miRNA seen in schizophrenia and its synaptic enrichment ratio observed in controls. Thus, synaptic miRNAs tended to be down-regulated in schizophrenia, and the more highly synaptically enriched miRNAs tended to show greater down-regulation. These findings point to some deficit in miRNA biogenesis, transport, processing or turnover in schizophrenia that is selective for the synaptic compartment. A novel class of ncRNA-derived small RNAs, shown to be strongly induced during an early phase of learning in mouse, is also expressed in man, and at least one representative (SNORD85) was strongly down-regulated in schizophrenia synaptosomes.

## Introduction

Profiling gene expression within human brain tissue is a basic starting point for understanding neuropsychiatric diseases. First, this information is critical for identifying specific genes and coordinated sets of genes that are altered in disease – these include not only genes which show altered expression due to genetic causes (e.g., SNPs or CNVs), but those which reflect altered pathways that contribute to pathogenesis (e.g. synaptic signaling, transcription factors, or epigenetic regulation). Second, this information serves as the basis for new unbiased approaches to diagnosis that cut across traditional symptom-based diagnostic categories. To date, most gene expression studies have focused on the exons of protein-coding genes. However, microRNAs are regulators of gene expression and protein translation in many tissues, including brain; thus, it is imperative to catalog the expression of miRNAs and other small RNAs in key regions of human brain as well as in major neuropsychiatric diseases. This may lead to new biomarkers for diagnostic subtyping and treatment response, as well as new therapeutic targets and new clues regarding etiology.

Several classes of noncoding RNAs (ncRNAs) have already been characterized and found to be important in regulating mRNA translation, stability, transcription, and/or epigenetic repression. These include microRNAs (miRNAs), endogenous siRNAs, piRNAs, pre-miRNAs, long intergenic noncoding RNAs (lincRNAs), antisense RNAs (asRNAs), and promoter-associated RNAs, among others. A relatively high proportion of miRNAs expressed in human brain are human- or primate-specific [1], [2]. In addition, nearly all types of abundant cellular ncRNAs such as tRNAs, rRNA, snoRNAs, vault RNAs and Y RNAs give rise to discrete processed tiny or small RNAs, and several of these have been shown to be processed by specific enzymes and to regulate specific RNA targets (e.g., [3]).

Furthermore, the machinery for miRNA biogenesis is associated with postsynaptic densities near synapses [4]–[6], and a subset of miRNAs (as well as antisense RNAs and other ncRNAs) are expressed and highly enriched in purified synaptic fractions [5], [7]. Mice and rats exposed to situations which alter synaptic activity show compartmentalized changes in miRNA expression within synaptic fractions [8]–[10]. Thus, it is important to measure ncRNAs not only in whole brain tissue, but specifically within the synaptic fraction as well.

The dorsolateral prefrontal cortex (BA 10) is chosen here for detailed study because it is involved in executive function and memory; is particularly well characterized in both basic, neuropathological and clinical studies; and shows discrete deficits relating to cognitive and behavioral phenotypes in a wide range of neuropsychiatric diseases including autism, depression and schizophrenia. Moreover, this region is evolutionarily one of the most advanced in the human species, and so may be expected to be a site in which human-specific ncRNAs play an important role in regulating neuronal functions.

At least seven recent studies have profiled miRNA levels in cerebral cortex in subjects dying with a diagnosis of schizophrenia or bipolar disorder [11]–[17], and several additional studies have examined genetic polymorphisms or miRNA-mRNA network correlations related to schizophrenia [18]–[22]. We have also previously characterized microRNA expression in a cohort of depressed suicide subjects [23]. In the present report, we have characterized microRNA expression in samples of prefrontal cortex (BA10) obtained from the Stanley Neuropathology Consortium consisting of well-characterized postmortem brain tissue from 15 adult subjects dying with a diagnosis of major depression, 15 with bipolar disorder, 15 with schizophrenia and 15 non-psychiatric controls [24]. We also identified changes that correlated with death by suicide across diagnostic categories.

## Materials and Methods

### Human Subjects

The Stanley Neuropathology Consortium consists of well-characterized postmortem brain tissue from 15 adult subjects dying with a diagnosis of major depression, 15 with bipolar disorder, 15 with schizophrenia and 15 non-psychiatric controls. The demographics and patient characteristics have been described previously [24] and many investigators have published findings using samples from this cohort. We obtained samples of frozen prefrontal cortex stored at −80C (Brodmann area 10) that spanned all cortical layers, and minced the tissue just prior to RNA isolation so that all layers would be represented in our samples. The research comes under exemption 4 and is approved by the IRB of the University of Illinois at Chicago.

### Experiment 1: RNA Isolation and miRNA Assay

PFC tissues were homogenized and total RNA isolated as described in [5], [23]. OD 260/280 values were 1.95–2.09. Total RNA was run on a denaturing agarose gel to confirm its integrity. RNA yields among the 4 groups were very similar. 1 microgram of total RNA was used for each sample, following the manufacturer’s protocol (Applied Biosystems, Inc./Life Technologies, Grand Island, NY, USA). The TLDA platform version 2.0 measures miRNA expression over two different 384-well plates; we employed the A plate which measures most (though not all) of the well-studied, relatively abundant miRNAs. RT-PCR assays and plate reading were carried out in multiples of 4 samples (one in each diagnostic group) to minimize technical variation. Most samples were run once, but several samples were run twice as technical duplicates and the Ct values were averaged.

### Data Cleansing

Examination of miRNA variability led to removal of 7 individual measurements as outliers (defined as measurements which were >3 SD different from any of the other measurements and observed in only a single sample). These did not affect any of the results described here. Two samples were removed from consideration as unsuitable (one bipolar and one schizophrenia sample, both suicides) because they exhibited far more undetectable miRNAs than the other samples. Technical variability of the TLDA plate might have reduced our ability to detect true differences across groups for certain individual miRNAs. For example, hsa-mir-155 and hsa-mir-23a (that were robustly expressed in previous studies [23] and in deep sequencing datasets) were undetectable in all samples in version 2.0 plates. In addition, certain miRNAs were well expressed in most samples but showed undetectable Ct values in 2–5 samples (sometimes including samples in the control group). By examining individual samples that were examined both by TLDA and deep sequencing, it appears that at least some of these “dropped” values are TLDA-related technical artifacts. Of the miRNAs that exhibited low values in one or more samples, only mir-181a and mir-181c showed significant differences across diagnostic groups; their measurements were regarded as suspect (and not shown in the Tables) since statistical significance was not achieved if the “dropped” values were omitted.

### Data Normalization and Statistical Analysis

It is common to normalize miRNA measurements using U RNAs as normalizers. However, we found that neither U6, U44, U48, nor the geometric mean of all three RNAs were equal and balanced across the four diagnostic groups, and so U RNAs could not be used as unbiased normalizers when looking for possible changes in expression in schizophrenia, bipolar disorder or depression. Instead, median normalization was employed: i.e., miRNA values in each sample were normalized to the median Ct of all expressed miRNAs in that sample. This method of normalization detects changes in individual miRNAs relative to others, but cannot detect global changes in the abundance of the miRNA population as a whole. On the other hand, U RNA expression was balanced across the pooled all-suicides vs. pooled non-suicide groups, so it was possible to examine this comparison for possible global changes.

As a screening procedure to identify miRNAs showing altered expression across groups, the t-test was performed (unpaired, two-tailed with equal variance since variances did not vary systematically by group) and miRNAs satisfying p<0.05 were tabulated. Those that satisfied this criterion were also assessed using the non-parametric Mann-Whitney U test (Table 1). We also identified miRNAs that showed alterations of mean expression of >40% above or below the control mean, regardless of p-value. There is value to all three ways of looking at the data. In general, we cannot assume that miRNA expression across individuals will follow a normal distribution; the Mann-Whitney test is more appropriate when miRNA expression does not satisfy a normal distribution, but is more conservative than the t-test when they do follow a normal distribution. Fold-change criteria were more useful when looking for patterns of change that were shared across diagnostic groups.

**Table 1:** miRNAs significantly altered in schizophrenia PFC.

Schizophrenia	fold-change	t-test p-value	M–W p-value	syn ratio
**miR-17-5p**	1.22	0.004	0.004	0.74
miR-331-5p	1.31	0.011	0.013	1.27
miR-16-5p	1.19	0.015	0.026	0.46
miR-187-3p	0.75	0.016	0.021	2.58
**miR-106b-5p**	1.22	0.018	0.032	0.91
**miR-485-5p**	0.7	0.02	0.013	1.78
miR-129-2-3p	0.74	0.021	0.029	1.56
miR-454-3p	1.21	0.033	0.049	1.06
miR-185-5p	1.22	0.035	0.021	1.62
miR-429-3p	1.34	0.035	0.045	1.65
miR-511	0.51	0.036	0.011	1.57
miR-18a-5p	1.28	0.038	0.045	1.29
miR-590-5p	1.4	0.039	0.067	0.71
miR-106a-5p	1.14	0.04	0.023	0.18
**miR-145-5p**	0.68	0.041	0.089	0.18
miR-642a-5p	1.89	0.044	0.014	0.98
miR-625-5p	1.34	0.044	0.049	1.22
**miR-508-3p**	0.48	0.045	0.045	1.52
miR-219-2-3p	1.48	0.049	0.049	0.88

Shown are miRNAs (Experiment 1) which differed from controls by t-test at p<0.05. The miRNAs that were altered in more than one diagnostic group (cf. Table 2) are shown in bold. The synaptic enrichment ratio was calculated from deep sequencing data (Experiment 2) based on its most abundant expressed sequence. Statistical significance p-values are also shown for the non-parametric Mann-Whitney (M–W) U test.

**Experiment 2: Illumina small RNA sequencing** was carried out on 6 representative control PFC samples (3 males and 3 females) as well as purified synaptosomes (prepared from a pool of the 3 males). Total RNA (1 microgram) was sequenced and analyzed as described in [25]), except that bar-coding was used to run all samples in parallel on sequencing lanes, and small RNAs were captured in the size range of 15–35 nt. cDNA library construction and deep sequencing were performed under the supervision of Alvaro G. Hernandez at the W.M. Keck Center, Univ. of Illinois at Urbana-Champaign, and raw sequences were prepared for bioinformatics analysis (adaptor trimming, quality filtering, and genome alignment) by Christopher J. Fields at the Institute for Genomic Biology, Univ. of Illinois at Urbana-Champaign.

**Human synaptosome preparation and characterization** followed the same protocol as for mouse tissue [4]–[6]

**Experiment 3: Illumina small RNA sequencing** was carried out on human synaptosomes (see Results) as described in [25], except that bar-coding was employed and small RNAs were captured in the size range of 15–40 nt. The small RNA libraries were prepared with Illumina’s TruSeq Small RNA Sample Prep kit. The libraries were quantitated by qPCR, and sequenced on one lane for 101 cycles on a HiSeq2000 using a TruSeq SBS sequencing kit version 3 and analyzed with Casava 1.8.2. The 3′-adaptor sequence was smallRNA-TGGAATTCTCGGGTGCCAAGGAACTCCAGTCAC. Deep sequencing data are noisier than TLDA data (counts for the same miRNA sequence measured in technical replicates often varied by 20%), and there were only 5 pooled samples per group (vs. 14–15 individual samples per group in the TLDA assay). Therefore, to screen for differential expression we employed nominal two-tailed t-tests with unequal variance (which is more conservative than equal variance), and counted a miRNA as showing significant changes only if its most abundant sequence was altered at p<0.05, and if the sum of all overlapping sequences in the 20–24 nt. size range was also altered at p<0.05

All data described in this paper have been deposited into the Stanley Neuropathology Consortium Integrative Database, http://sncid.stanleyresearch.org/.

## Results and Discussion

### Experiment 1: Measuring miRNA expression across the Stanley Neuropathology Consortium using high-throughput RT-PCR plates

#### Results

A total of 377 miRNAs were assayed on the TLDA A plate, together with U6, U44 and U48 regarded as housekeeping genes. Of these, 232 miRNAs showed robust expression (mean Ct value ≤34 in at least one diagnostic group, i.e., ≥2-fold more abundant than the threshold value of 35) (File S1).

#### a) Alterations in schizophrenia, bipolar and depression

Because U RNAs were not suitable for normalizing expression values across diagnostic categories, median normalization was employed instead. Note that median normalized data identify miRNAs whose expression is altered relative to other miRNAs in the population, and do not detect changes in absolute expression level (e.g. when the entire population of miRNAs is shifted up or down). As shown in Table 1, a total of 19 miRNAs showed altered expression (13 up, 6 down) in schizophrenia subjects by t-test. Similarly, a total of 9 miRNAs were significantly altered (4 up, 5 down) in bipolar subjects (Table 2). In contrast, only two miRNAs showed significant changes in the depression group at p<0.05 (mir-508-3p and mir-152, both down-regulated). Interestingly, mir-508-3p was strongly down-regulated in all three diagnostic groups, being significant in schizophrenia (p = 0.045) and depression (p = 0.01) but just missing significance in the bipolar group (p = 0.07) (Tables 1, 2, 3). This miRNA is noteworthy because it is primate-specific, one of a cluster of primate-specific miRNAs encoded on the X chromosome that are predominantly expressed in testes.

**Table 2:** miRNAs significantly altered in bipolar disorder and major depression PFC.

Bipolar	fold-change	t-test p-value	M–W p-value
**miR-17-5p**	1.23	0.0028	0.0012
**miR-145-5p**	0.63	0.0069	0.016
miR-579	2.06	0.0092	0.013
**miR-106b-5p**	1.18	0.021	0.029
**miR-485-5p**	0.55	0.036	0.061
miR-370	0.85	0.041	0.061
miR-500a-5p	0.84	0.041	0.04
miR-34a-5p	0.85	0.048	0.074
miR-29c-3p	1.55	0.049	0.11
**Depression**			
**miR-508-3p**	0.43	0.011	0.018
miR-152-3p	0.799	0.016	0.012

Shown are miRNAs (Experiment 1) which differed from controls by t-test at p<0. The miRNAs that were altered in more than one diagnostic group (cf. Table 1) are shown in bold. Statistical significance p-values are also shown for the non-parametric Mann-Whitney (M–W) U test.

**Table 3:** miRNAs altered by 40% or more in one or more diagnostic groups in PFC.

Schizophrenia	fold-change	p-value
miR-197	0.46	0.15
**miR-508-3p**	0.48	0.045
miR-511	0.51	0.035
miR-221	0.51	0.44
miR-93	1.4	0.19
**miR-219-2-3p**	1.48	0.049
**miR-579**	1.55	0.076
miR-338-3p	1.7	0.059
**miR-219-5p**	1.71	0.07
**miR-218**	1.86	0.35
**miR-642**	1.89	0.044
**Bipolar**		
**miR-508-3p**	0.53	0.07
miR-485-5p	0.55	0.036
**miR-219-2-3p**	1.41	0.11
miR-886-3p	1.42	0.28
**miR-219-5p**	1.52	0.18
miR-29c	1.55	0.049
**miR-642**	1.6	0.17
**miR-221**	1.67	0.33
**miR-210**	1.86	0.18
**miR-218**	1.94	0.32
**miR-579**	2.06	0.0093
**Depression**		
miR-199a-3p	0.42	0.095
**miR-508-3p**	0.43	0.012
miR-330-3p	0.58	0.23
miR-499-5p	1.44	0.23
miR-200c	1.52	0.1
miR-671-3p	1.56	0.14
**miR-210**	1.6	0.4
miR-134	1.65	0.17
**miR-221**	1.69	0.32
**miR-218**	1.89	0.33

miRNAs up- or down-regulated by 40% or more relative to controls (Experiment 1). Those showing similar extent and direction of change in two or more groups are bolded. P-value refers to statistical significance by t-test.

**Figure 1:**
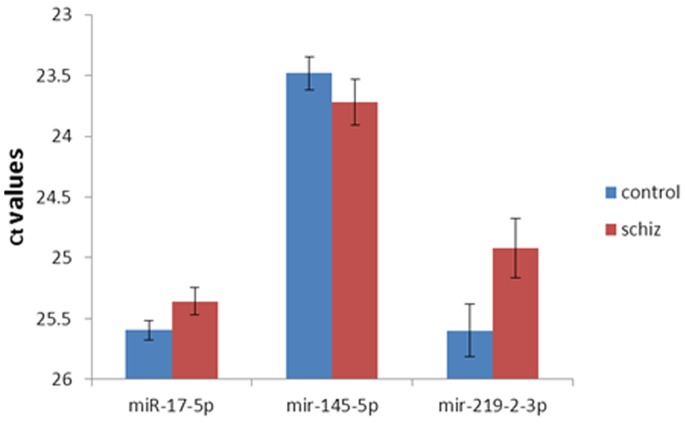
Manual RT-PCR measurements of 3 selected miRNAs agree very well with measurements performed by TLDA assay. The control and schizophrenia samples of Experiment 1 were measured using individual TaqMan primer sets for miR-17, miR-145-5p and miR-219-2-3p. Note that lower Ct values indicate higher abundance on a log2 scale (i.e., a decrease of 0.5 Ct represents a 41% increase in abundance or 1.41-fold difference across groups). Error bars indicate standard error of the mean. In this assay, miR-17-5p showed a 1.18-fold increase (p = 0.05), which is very close to 1.22-fold reported in Table 1. miR-145-5p showed a 0.85-fold change (p = 0.16), similar to 0.68-fold in Table 1. miR-219-2-3p showed a 1.60-fold increase (p = 0.029), similar to 1.48-fold in Table 1.

The lists of individual miRNAs that achieved nominal statistical significance (Tables 1,2, 4) should not be over-interpreted, since for any set of 232 miRNA measurements, one would expect about 6 miRNAs to be apparently elevated and 6 to be decreased just by chance (at p = 0.05). Nevertheless, we measured the fold-changes of three selected miRNAs listed in Table 1 by manual RT-PCR and found that the fold-changes agree very well with those measured by TLDA assay (Figure 1). This suggests that the TLDA assay measures the direction and magnitude of miRNA changes in a reliable manner.

**Table 4:** miRNAs altered in suicide.

all suicides	fold-change	p-value
**miR-152**	0.79	0.00059
**miR-181a**	0.34	0.0105
**miR-330-3p**	0.53	0.012
**miR-34a**	0.85	0.016
**miR-224**	0.57	0.036
miR-376a	1.23	0.037
**miR-133b**	0.81	0.04
miR-625	1.25	0.045
**up- or down-regulated by 40% or more:**		
miR-330-3p	0.53	0.0125
miR-224	0.57	0.036
miR-579	1.43	0.059
miR-34c-5p	1.54	0.097

The group of all suicides (across diagnostic categories) was compared to all non-suicides (Experiment 1). miRNAs showing significant changes by t-test at p<0.05 are listed. Bolded miRNAs were also significantly altered when data was normalized to the geometric mean of U6, U44 and U48. Separately, miRNAs showing changes of >40% or greater are listed.

Rather than viewing the miRNAs as candidates to be validated individually, we focused on ascertaining large-scale patterns, both across all expressed miRNAs, and across the set of nominally affected miRNAs. Note that about half of the miRNAs altered in bipolar samples showed changes that were similar in extent and direction as in schizophrenia. Indeed, the same up-regulated miRNA, mir-17-5p, exhibited the strongest level of statistical significance in both groups (Tables 1 and 2). Across all expressed miRNAs, the fold-changes in bipolar disorder were significantly correlated to those in schizophrenia (r = 0. 591; p<0.0001), and separately to depression (r = 0.50; p<0.0001). Although fold-changes in depression were also correlated to those in schizophrenia (r = 0.31, p<0.0001), multiple regression analysis indicates that these two groups are not related significantly once the changes in bipolar disorder are factored out (r = 0.018; p = 0.79). Shared changes across diagnostic groups were even more obvious when considering the set of miRNAs that showed alterations of 40% or greater (regardless of statistical significance level). As shown in Table 3, more than half (6 of 11) of the miRNAs altered more than 40% in schizophrenia showed similar extent and direction of change in bipolar disorder, while more than a third (4 of 10) of miRNAs altered in depression showed similar alterations in bipolar disorder.

To look for patterns in the set of miRNAs altered in one or more of the diagnostic groups, we examined the set of miRNAs in Tables 1 and 2 for shared precursor transcripts and shared 5′-seed sequences. Only two of these miRNAs derived from the same primary miRNA gene transcript (mir-17 and mir-18a), showing similar extent of up-regulation in schizophrenia in both cases. More impressive was the number of miRNAs who shared seed sequences: The 5′-seed for mir-17-5p, AAACUGC, is shared with two other miRNAs, mir-106a and mir-106b, and AAACUGC is also found near the 3′-end of mir-590-5p, which was identified as a probable 3′-seed sequence [26]. All of these are up-regulated in schizophrenia (and none are especially enriched in brain). Intriguingly, all five of these miRNAs (17-5p, 18a, 106a, 106b, and 590-5p) also contain CAGUGCA or AGUGCA within the 3′-half of their sequences. AGUGCA is expressed in mir-152 and numerous other human miRNAs, both within 5′seeds and towards the 3′half of the sequence, and possibly may represent a functional recognition or targeting motif regardless of its particular placement. Since 5′-seeds and 3′-seeds are important for selection of mRNA targets, this subset of up-regulated miRNAs could target a similar or overlapping set of mRNA targets.

In contrast to this group of up-regulated miRNAs, the set of miRNAs down-regulated in schizophrenia followed a quite different pattern, which will be discussed under Experiments 2 and 3, below.

#### b) Comparison of all-suicide vs. all non-suicide subjects

About half of the subjects in the disease groups died by suicide (8/14 bipolar subjects, 7/15 depressed subjects, 3/14 schizophrenic subjects and none of the controls). (One bipolar and one schizophrenia suicide sample were excluded from data analysis because of anomalously low miRNA expression.) When all suicide subjects were pooled together into one group regardless of diagnosis, 8 miRNAs were significantly altered (2 up, 6 down) using median-normalized data (Table 4). (Because U RNA expression was balanced across the pooled all-suicides vs. pooled non-suicide groups, it was also possible to examine miRNA expression using U RNA-normalization. We found significant changes in 11 miRNAs, all down-regulated, which overlapped extensively with the list produced using median normalization (Table 4).) Of the altered miRNAs, the most intriguing is mir-152 because it showed the strongest significance value regardless of how the data were normalized. miR-152 was highly significantly down-regulated in depression as well (Table 2), which is not trivial since less than half of the depressed subjects died by suicide, and less than half of the suicide subjects were in the depression group.

#### Discussion of Experiment 1

The Stanley Neuropathology Consortium is a popular benchmark for human postmortem studies, and its samples are well balanced on such parameters as age, gender, race, and brain pH [24]. However, it should be acknowledged that the diagnostic groups are not balanced for postmortem interval. For the 58 samples that we analyzed in this study, the PMIs (means±S.D.) are 24.7±9.2 hr in controls, 26.4±10.9 hr in depression, 31.6±15.7 hr in bipolar disorder, and 35.1±13.6 hr in schizophrenia; the values for the schizophrenia group are significantly different from controls (p = 0.031). This is likely the reason that U RNAs were not equally expressed across groups. Therefore, it is critical to consider whether differences in PMI might have confounded the miRNA expression data as well. However, five considerations provide compelling evidence that there was no overall loss of miRNA expression in the schizophrenia samples: a) Overall miRNA abundance was unchanged between controls and schizophrenia groups in Experiment 1, using both normalized and non-normalized data. b) Of the miRNAs that were significantly changed in schizophrenia, twice as many were up-regulated as down-regulated. c) We used median normalization of Ct values which removes any individual changes in overall miRNA abundance due to random degradation. d) Two samples which did show overt overall loss of miRNA abundance were excluded from analysis. e) Finally, we calculated RNA quality values for the control and schizophrenia samples that were assayed by TLDA, using leftover RNA (that had since been thawed several times). The RIN values of the control group (mean 5.5+/− s.e.m. 0.39) were not significantly different from the schizophrenia group (mean 4.9+/− s.e.m. 0.24; p = 0.22). Thus, we are confident that the miRNA measurements in Experiment 1 are not confounded by PMI differences (leading to differences in the extent of RNA degradation).

#### Comparison of the present results to previous studies

It is difficult to directly compare these data to previous studies, for several reasons. a) Different brain regions may be expected to have different functions and exhibit somewhat different alterations (possibly even for subfields of prefrontal cortex such as BA9 vs. BA10). b) Variable sampling of cortical layers and white matter may occur as well. c) The demographics of cohorts are not necessarily comparable. d) A wide variety of miRNA measurement platforms, and different methods of normalization, have been utilized. e) Finally, the usual practice of ranking and describing individual miRNAs in terms of statistical significance is not very robust. The same miRNA may exhibit the same fold-change in two studies, and be significant in one case yet not significant at all in the other. For example, Miller et al [14] observed down-regulation of mir-132 in their schizophrenia group (0.79-fold, which is comparable to 0.84-fold in our study) but not in their bipolar group (which agrees with the 0.99-fold change observed in our study). However, whereas this finding was statistically significant and highlighted in their dataset (p = 0.016), it was not in ours (p = 0.33).

According to a recent review of miRNA expression in schizophrenia [27], a total of 13 miRNAs that were measured in our TLDA assay have been associated with increased expression in multiple previous studies (miR-128a, miR-15a, miR-15b, miR-16, miR-17, miR-199a*, miR-20a, miR-222, miR-34a, miR-452*, miR-486, miR-487a, miR-652). Actually (disregarding the size or statistical significance of the changes), 9 of these 13 miRNAs showed some up-regulation in our median-normalized TLDA dataset. Conversely, 5 miRNAs showed decreased expression across multiple studies (miR-106b, miR-20b, miR-224, miR-30b, miR-383), of which 3 miRNAs showed some down-regulation in the TLDA dataset. These results suggest that meta-analyses of the raw data from accumulated studies should be a fruitful approach in identifying a core set of the most reliably observed changes in each disorder.

In our previous study, we had reported a global decrease in miRNA expression in a McGill cohort of depressed suicide subjects [23]. The global decrease was replicated here in the Stanley pooled suicide group: Across all expressed miRNAs, using U RNA normalization, 70% (165 of 237) were decreased in mean abundance in the suicide group, indicating that most miRs showed a global downward trend. On the other hand, mir-152, down-regulated significantly in the Stanley cohort (Table 2), did not show any large or significant change in expression in our previous study [23], which was performed in McGill cohort. It is not clear why the results differ in this manner, though subject characteristics may be a factor. The Stanley cohort [24] and the McGill cohort [23] are similar in mean age, PMI and brain pH, but there are several significant differences in demographics. Only half (7/15) of the Stanley subjects died by suicide whereas all of the McGill subjects died by suicide; 3 of the Stanley subjects had current alcohol/drug abuse or dependence at the time of death, whereas none of the McGill subjects did; and the gender balance was different (Stanley M/F = 9/6, McGill M/F = 16/2).

### Experiment 2: Deep Sequencing of Control PFC (Males and Females) and Purified Synaptosomes

A subset of miRNAs have been shown to be enriched within synaptic fractions (synaptosomes and synaptoneurosomes) isolated from mouse and rat forebrain relative to whole tissue [5]. The synaptically enriched miRNAs show biological, structural and evolutionary differences from non-enriched miRNAs [5], [7]. The essential components of miRNA biogenesis (including pri-miRs, drosha, DGCR8, dicer and Ago) are expressed locally within dendritic spines in close proximity to postsynaptic densities [4], [6]. Paradigms that stimulate synaptic activity cause changes in synaptic miRNA expression that are different than seen in whole tissue [8]–[10].

To characterize the synaptic enrichment profile of human miRNAs, we carried out deep sequencing in a number of control PFC samples (3 males and 3 females) as well as purified synaptosomes (prepared from a pool of the 3 males). Sequences which aligned exactly to the human genome, and which mapped to genomic loci corresponding to known microRNAs (miRBAse 18) were placed into a dataset for further examination (File S2). This allowed us to check the results of TLDA assays against deep sequencing and to estimate the synaptic enrichment of each miRNA.

#### a) Deep sequencing data vs. TLDA assays in whole PFC tissue

The primers and TaqMan probes used in the TLDA version 2.0 platform were derived from the canonical human miRNA sequences listed in miRBase version 14. As typically observed in deep sequencing datasets, most miRNAs were expressed as a mix of different sequence variants (File S2). In a significant minority (∼20%) of cases, the most abundant form of the miRNA expressed in human prefrontal cortex differed from the “official” canonical sequences listed in miRBase (versions 14 to 19, the most recent release). Most of these were 3′-end variants but about 5% of the time, the most abundant expressed sequence (by deep sequencing) was a 5′-variant relative to the miRBase sequence (e.g., mir-577, Table 5; see also [28]). Similar discrepancies were also observed in the synaptosome dataset (Experiment 3). This is important to acknowledge because RT-PCR based detection methods often depend critically on knowing the exact 3′-end of the mature miRNA, and miRNA target prediction algorithms often depend critically on knowing the exact 5′-end of the mature miRNA sequence. This is one reason that TLDA assays may potentially differ considerably from the expression values determined by deep sequencing. This also implies that TLDA assays may potentially be affected by changes in the relative proportions of miRNA variants even when the overall levels of a given miRNA do not change.

**Table 5:** miR-577 variants vary in 5′-end and male/female expression ratio.

Sequence	All	Males	Females	Syn	Syn ratio	M/F
**GTAGATAAAATATTGGTACCTG**	9951.3	8416	9196.2	4496.15	0.53	**0.92**
TAGATAAAATATTGGTACCTG	8021.5	2941.33	10489.19	1585.9	0.54	0.28
**GTAGATAAAATATTGGTACCT**	1577	1359.33	1436.81	853.5	0.63	**0.95**
TAGATAAAATATTGGTACCTGA	1329	367	1834.1	172.5	0.47	0.2
**GTAGATAAAATATTGGTACC**	434.5	412	365.87	213.8	0.52	**1.13**
**GTAGATAAAATATTGGTACCTGA**	208.66	148.66	215.09	89.02	0.6	**0.69**
TAGATAAAATATTGGTACCT	183.66	64.66	242.31	38.55	0.6	0.27
all GTA variants	12171	10336	11214		0.55	**0.92**
all TAG variants	9534	3373	12566		0.53	0.27

Shown are all of the mir-577 variants expressed in human PFC at >100 counts per sample (Experiment 2). Variants that begin with GTA, i.e. that have an extra 5′-base compared to the canonical miRBase entry for mir-577, are indicated in bold. Although variants beginning with GTA and those beginning with TAG were expressed at roughly comparable levels, and had similar synaptic enrichment ratios, the GTA variants were expressed almost equally in males vs. females whereas the TAG variants were expressed predominantly in females. Synaptic enrichment ratio was calculated as the normalized synaptosome counts/mean counts in males.

The deep sequencing dataset contained data that inherently could not be detected using the TLDA platform. For example, mir-577 (not assayed on the TLDA plate) is expressed as a set of 55 different sequence variants, 7 of which are expressed at an average of ≥100 reads per sample (Table 5). The most abundant sequence is GTAGATAAAATATTGGTACCTG, which contains an extra base at the 5′-end compared to the canonical miRBase 19 sequence TAGATAAAATATTGGTACCTG. Not only do these have different 5′-seed sequences (and hence would be predicted to prefer different targets), but the different 5′-variants had different expression according to gender. The variants beginning with GTA were all expressed roughly equally in males and females (M/F = 0.92), whereas those beginning TAG were all expressed predominantly in females (M/F = 0.26) (Table 5).

#### b) miRNA synaptic enrichment in human PFC

**Figure 2:**
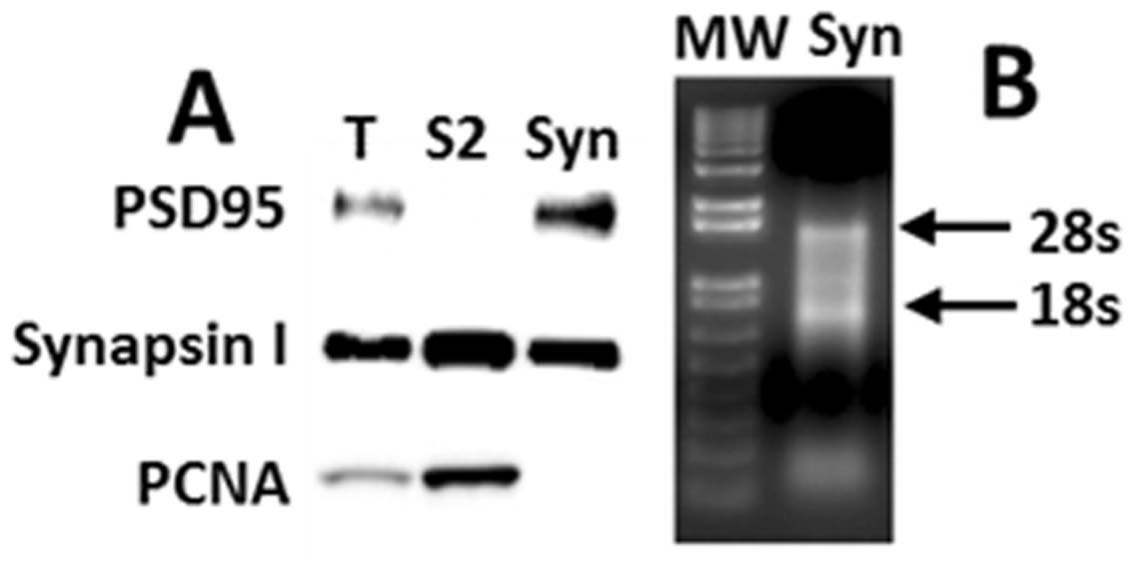
Human PFC sample processed for synaptosome isolation and examined for protein and total RNA. (A) Equal amount of proteins (12 µg) were subjected to PAGE and blotted with anti-PSD95 (Millipore mouse monoclonal clone 28/43; 1∶30,000), anti-Synapsin I (Chemicon AB1543P, lot #LV1354224, rabbit polyclonal affinity purified; 1∶30,000), or anti-PCNA (Sigma, P-8825, clone PC10, mouse monoclonal; 1∶1000) antibody. Fractions are: T, total homogenate; S2, 20,000×20 min supernatant; Syn, synaptosomes. The raw data are shown in File S5. (B) Synaptosomal RNA (0.5 µg) was loaded in a 0.9% TBE Agarose gel and stained with Ethidium Bromide. MW is the 1 Kb plus ladder size markers.

Human synaptosomes were similar to synaptosomes prepared from fresh mouse brain, in terms of enrichment in synaptic protein markers (e.g. fig. 2) and RNA markers (e.g. Camk2a mRNA, not shown), and depletion of nuclear markers (e.g. PCNA, fig. 2). The median synaptic enrichment ratio across all miRNAs was 1.01, with 15% of sequences showing enrichment >1.5-fold (6% >2-fold) and 17% showing depletion >1.5-fold (7% ≥2-fold). The range of synaptic enrichment ratios varied from ∼5-fold enriched in synaptosomes relative to whole tissue (mir-219-5p) to ∼5-fold depleted (mir-145). This dynamic range was similar to that reported previously in mouse hippocampus [5]. The estimated synaptic enrichment ratios for each miRNA sequence read are shown in File S2.

Using the synaptic enrichment estimates from Experiment 2, we re-assessed the findings of Experiment 1, and noted that 5 of the 6 miRNAs that were down-regulated significantly in the schizophrenia group had synaptic enrichment ratios of 1.5 or greater, whereas only 2 of the 13 up-regulated miRNAs were synaptically enriched to that extent (Table 1). Conversely, 5 of the up-regulated miRNAs (and none of the down-regulated miRNAs) were listed in Wang et al. as being expressed predominantly in white matter [29]. This raised the possibility that the down-regulation of miRNAs might be related to the synaptic compartment. In order to test this directly, we prepared synaptosomes from the control vs. schizophrenia groups and examined miRNA expression via Illumina deep sequencing.

### Experiment 3: Deep Sequencing of Human PFC Synaptosomes in Controls vs. Schizophrenia

#### Results

Synaptosomes were prepared from schizophrenia vs. controls (5 pools of 3 individuals in control group and 6 pools of 2–3 individuals in schizophrenia group) and subjected to Illumina deep sequencing in the range from 15–40 nt. Sequences were aligned using Novoalign to the human genome (hg 19) and those which aligned exactly (uniquely or to multiple loci) were analyzed (Files S3 and S4). Separately, we aligned sequences directly against miRBase 19 hairpin and mature sequences, allowing for some mismatches to detect edited or non-genomically tailed sequences (not shown). These different mapping strategies gave slightly different sets of sequences, but results were similar in both cases. A few miRNAs were detected by TLDA that were expressed at extremely low levels by deep sequencing and not contained in our filtered dataset (e.g. mir-508-3p, mir-511). Of the 4 subjects in the schizophrenia group who died by suicide, one sample was excluded because it had poor miRNA expression and the other 3 were placed together in a single synaptosome pool. Because suicide may affect miRNA expression (see Experiment 1 and [23]), and since including this synaptosome pool slightly decreased the overall level of significance in the dataset as a whole (not shown), the suicide subjects were excluded from the present analyses.

The synaptosomal RNAs exhibited a major peak at 20–22 nt. corresponding to miRNAs, but expressed sequences throughout the entire range of 15–39 nt. (Table 6). When the total number of expressed RNAs were compared by t-test for each sequence length, there was a selective, significant loss of expression in schizophrenia only for certain sequence lengths (22, 23 and 25 nt.) that lie at the upper end of the miRNA range (Table 6).

**Table 6:** Distribution of sequence counts by length in control vs. schizophrenia synaptosomes.

Length	# reads in controls	# reads in schizophrenia	#unique sequences
15	17983.29	12971.43	238
16	27108.04	20873.8	371
17	27617.05	24289.14	625
18	52008.46	51611.2	903
19	101094.2	97008	1189
20	250731.2	235637.7	1462
21	1268842	1155849	1570
**22**	**983249.7**	**754138.3**	**1500**
**23**	**281887.1**	**200128.3**	**1033**
24	296062.3	252843.7	531
**25**	**19364.15**	**13119.82**	**402**
26	8131.598	6826.838	369
27	15757.37	15823.5	491
28	13304.94	13097.21	482
29	18915.51	28155.49	528
30	8593.995	9005.574	494
31	10049.84	10067.87	535
32	15124.49	16015.68	496
33	22231.07	19894.17	511
34	13358.98	15989.51	531
35	48970.03	48429.35	671
36	25953.74	37097.25	651
37	33611.33	46538.93	730
38	15750.05	23834.14	823
39	23704.65	44615.48	763

5 samples of pooled control synaptosomes were compared to 5 pools prepared from schizophrenia synaptosomes (Experiment 3). Values represent the average number of sequences per sample that aligned to the human genome within miRNAs or other known transcript loci, normalized as raw counts per million mappable reads. Bolded values are significantly different from control (using summed values for each pooled sample, using two tailed t-test, p<0.05). Number of unique reads refers to the number of distinct sequences in the entire dataset.

Individual miRNAs were examined for differential expression, looking for mature miRNAs satisfying both of the following criteria: a) its most highly abundant expressed sequence was altered significantly at p<0.05 by t-test, and b) the sum of all overlapping sequences at that site (in the size range of 20–24 nt.) were also altered significantly at p<0.05 by t-test. Using this criterion, 73 miRNAs were down-regulated, whereas only one was (marginally) up-regulated (mir-96-5p, up 2.14-fold, p = 0.028 for its most abundant sequence but p = 0.082 for sum of all overlapping sequences; note that mir-96-5p has a very low synaptic enrichment ratio = 0.18).

Strikingly, across all expressed miRNAs in synaptosomes, there was a highly significant inverse correlation (r = −0.415; two-tailed p<0.0001) between the fold-change of a given miRNA sequence seen in schizophrenia (Experiment 3) and its synaptic enrichment ratio observed in controls (Experiment 2). This down-regulation cannot be explained by loss of synapses (or loss of RNA) in schizophrenia, since sequencing produced a similar total number of mappable reads in each group. (Approximately 1 microgram of total RNA was sent for sequencing from each synaptosome pool, except for one sample in the schizophrenia group that had about 0.44 microgram. However, even in this sample, the number and percentage of mappable reads was similar to the other samples (File S3, sheet 1).

Instead, taken together, these findings point to some deficit in miRNA biogenesis, transport, processing or turnover that is selective for the synaptic compartment. In this context, it is interesting that down-regulating miRNAs selectively in excitatory neurons, in mouse forebrain, produces discrete deficits in short-term plasticity and inhibitory neurotransmission that are reminiscent in some ways of a schizophrenia phenotype [30], [31].

Finally, we examined several potential confounds that might have artifactually led to real or apparent global down-regulation of miRNA expression in this study. For example, the miRNA decrease could potentially be an artifact related to normalization method. Global effects might also be caused by possible ceiling effects – that is, if a nonspecific increase in RNA degradation fragments occurred in the schizophrenia samples, this might force a decrease in other sequenced small RNAs in the same prep. To examine these possibilities, we looked at the subset of miRNA sequences that showed the biggest decrease in schizophrenia, i.e. those having 22 or 23 nt length (Table 6). The normalized counts in the schizophrenia group were, on average, 72.7% of control values (p = 0.016). In contrast, when we collected all H/ACA and C/D box snoRNA sequences of the same length, the normalized snoRNA counts in schizophrenia were 102% of control values (p = 0.92), i.e. not decreased or affected at all. Thus, the decreases were not related to sequence length per se, but specifically to miRNAs.

We also confirmed that miRNA counts in the samples were not systematically affected by changes in PMI. Again, we focused on the set of miRNA sequences having 22–23 nt length, and correlated the total number of sequences in each pooled sample with the average or maximal PMI of the samples comprising each pool. The correlation with average PMI was r = 0.24, i.e., a small (and positive) relationship, whereas the correlation with maximal PMI was r = 0.08, essentially zero. Thus, decreased miRNA expression levels could not be explained by PMI differences across samples.

#### Other small RNAs

Besides miRNAs, a variety of other small RNAs detected in human PFC tissue was also detected in synaptosomes. Although a detailed analysis of these RNAs lies beyond the scope of the present paper, a handful of novel small RNAs were both well-expressed in human synaptosomes and showed significant changes in schizophrenia.

For example, a set of sequences derived from a C/D box snoRNA, SNORD85, showed 50% decrease in schizophrenia synaptosomes (Table 7). The most abundant of these, TTCACTGATGAGAGCATTGTTCTGAGC, is a 27 mer that encompasses a C/D box and terminates 4 bases before the 3′end of the host snoRNA. This closely resembles the features of the novel class of ncRNA-derived small RNAs described in mouse that are strongly induced during an early phase of 2-odor olfactory discrimination training, but not induced in two control groups [25], [32]. The observed down-regulation in schizophrenia was restricted to sequences in the size range of 25–30 nt, which is the size range that was selectively induced by learning in mice. (Other snoRNA-derived small RNAs were expressed that were shorter or longer than this range, but were not affected comparably neither in learning nor in schizophrenia; Table 7). This is the first evidence that this novel class of small ncRNAs is expressed in man (and in synaptosomes), and at least one representative of this class is significantly altered in a neuropsychiatric disorder. This is exciting because it could potentially point to a deficit in a novel plasticity pathway involving ncRNAs.

**Table 7:** SNORD85-derived small RNAs that are down-regulated in schizophrenia synaptosomes.

SNORD85 derived sequences	length	Schiz	control	fold-change	p-value
TGGCAAGGATGATACAC	17	15.9	10.3	1.54	0.29
TGGCAAGGATGATACACA	18	13.5	13.4	1.01	0.98
TGGCAAGGATGATACACACT	20	23.5	15.1	1.55	0.31
TCACTGATGAGAGCATTGTTCTGAGC	26	4.98	15.38	0.32	**0.0053**
TTCACTGATGAGAGCATTGTTCTGAG	26	8.4	11.27	0.75	0.43
TTCACTGATGAGAGCATTGTTCTGAGC	27	121.8	220.2	0.55	0.069
GTTCACTGATGAGAGCATTGTTCTGAGC	28	40.3	101.7	0.396	**0.0096**
TTCACTGATGAGAGCATTGTTCTGAGCCA	29	4.08	10.1	0.4	**0.0048**
TTCACTGATGAGAGCATTGTTCTGAGCCAG	30	8.96	11.28	0.79	0.44
**sum of all 25–30 nt. reads**		188.52	369.93	0.51	**0.037**
TGGCAAGGATGATACACACTTGCCCTCACTTAGACT	36	24.6	11.17	2.2	0.21
TGGCAAGGATGATACACACTTGCCCTCACTTAGACTA	37	36.28	18.78	1.93	0.34

Small RNAs are shown that aligned to the C/D box SNORD85 locus with >10 sequence reads (normalized) per sample in either the control or the schizophrenia group. Shown are average counts for schizophrenia group, control group, as well as the fold-change. Those that are significantly altered by t-test at p<0.05 are shown in bold.

Other significant changes in non-miRNA small RNAs will be mentioned briefly (Table 8): **a)** A set of small RNAs derived from Y3 RNAs was strongly down-regulated in schizophrenia (50% decrease). Changes were restricted to sequences in the 23–25 nt. size range. Y RNA-derived small RNAs form in cells in a regulated fashion (up-regulated by poly (I:C) treatment and in a variety of tumors) and Y RNAs have a stem-loop structure reminiscent of pre-miRNAs, but their function is not clear. At least some Y RNA fragments do not bind to Argonaute2 [33] but some Y RNA-derived small RNAs bind to Miwi and regulate dendritic spine morphogenesis [34]. **b)** A few altered RNAs mapped to genomic loci lacking known processed transcripts. For example, a set of overlapping short sequences (15–19 nt.) mapped uniquely to an intron of PEAK1 (lacking any annotation regarding ncRNAs encoded in this region), but were well-expressed (200 sequence reads per control sample) and were strongly down-regulated in schizophrenia (73% decrease). **c)** Some sequences were up-regulated – for example, mitochondrial RNA processing endoribonuclease (RMRP)-derived small RNAs (all 32–39 nt.) were significantly up-regulated (2.23-fold). These data will be described in more detail elsewhere, but they illustrate the importance of examining the full set, and full size range, of expressed small RNAs.

**Table 8:** Non-miRNA small RNAs that are altered in schizophrenia synaptosomes.

PEAK1 derived sequences	length	Schiz	control	fold-change	p-value
CATTCAAAAAAGAGTACC	18	134.8	196.97	0.27	**0.045**
TTCAAAAAAGAGTACC	16	47.4	59.5	0.3	**0.026**
ACATTCAAAAAAGAGTACC	19	35.3	32.9	0.34	**0.054**
**RMRP derived sequences**					
GGTTCGTGCTGAAGGCCTGTATCCTAGGCTACACACTG	38	230.3	88.9	2.59	**0.032**
GTGGTTCGTGCTGAAGGCCTGTATCCTAGGCTACACAC	38	336.2	218.3	1.54	0.23
GGTTCGTGCTGAAGGCCTGTATCCTAGGCTACACACT	37	9	3.5	2.57	**0.015**
AGGCCTGTATCCTAGGCTACACACTGAGGACT	32	3.3	0.6	5.3	0.14
CGTGCTGAAGGCCTGTATCCTAGGCTACACACTG	34	18.4	4.7	3.92	0.11
TGGTTCGTGCTGAAGGCCTGTATCCTAGGCTACACAC	37	75.9	41.8	1.82	**0.04**
GTTCGTGCTGAAGGCCTGTATCCTAGGCTACACACTG	37	42.5	15.1	2.82	0.077
GGTTCGTGCTGAAGGCCTGTATCCTAGGCTACACAC	36	19.9	12.4	1.61	0.06
GTGCTGAAGGCCTGTATCCTAGGCTACACACTG	33	10.9	2.3	4.7	0.17
TGAAGGCCTGTATCCTAGGCTACACACTGAGGACT	35	5.2	0.6	8.44	0.24
GTTCGTGCTGAAGGCCTGTATCCTAGGCTACACACT	36	2.9	0.8	3.49	0.16
TCGTGCTGAAGGCCTGTATCCTAGGCTACACACTG	35	38.1	6.4	5.99	0.24
GAAGGCCTGTATCCTAGGCTACACACTGAGGACT	34	1.9	0		0.29
TGCTGAAGGCCTGTATCCTAGGCTACACACTGAGGACT	38	38.5	8.3	4.64	0.28
TTCGTGCTGAAGGCCTGTATCCTAGGCTACACACTG	36	29.2	3.3	8.92	0.28
CTGAAGGCCTGTATCCTAGGCTACACACTGAGGACT	36	11.6	0.7	16.78	0.31
GCTGAAGGCCTGTATCCTAGGCTACACACTGAGGACT	37	51.5	2.4	21.18	0.33
GGTTAGTGCTGAAGGCCTGTATCCTAGGCTACACACTG	38	2.9	1.4	2.18	0.26
TGGTTCGTGCTGAAGGCCTGTATCCTAGGCTACACACTG	39	3.7	2.4	1.53	0.6
GTTCGTGCTGAAGGCCTGTATCCTAGGCTACACAC	35	11.2	8	1.39	0.42
TCGTGCTGAAGGCCTGTATCCTAGGCTACACACT	34	1.7	0.9	2.03	0.51
**sum of all RMRP sequences**		944.8	422.8	2.23	**0.043**
**Y3 derived sequences**					
GGCTGGTCCGAGTGCAGTGGTGT	23	59.5	128.6	0.46	**0.034**
GGCTGGTCCGAGTGCAGTGGTGTT	24	68	137	0.5	**0.014**
GGCTGGTCCGAGTGCAGTGGTGTTT	25	192.6	321.3	0.6	**0.05**
GGCTGGTCCGAGTGCAGTGGTGTTTACA	28	94.2	149.2	0.63	0.23
GGCTGGTCCGAGTGCAGTGGTGTTTACAAC	30	795.3	496.7	1.6	0.3
GGCTGGTCCGAGTGCAGTGGTGTTTACAACT	31	1669.8	571.6	2.92	0.36
GGCTGGTCCGAGTGCAGTGGTGTTTACAACTA	32	574	300.4	1.91	0.22

Small RNAs are shown that aligned to PEAK1, RMRP or Y3 RNA (within DEAK1 locus) with >10 sequence reads (normalized) per sample in either the control or the schizophrenia group. Shown are average counts for schizophrenia group, control group, as well as the fold-change. Those that are significantly altered by t-test at p<0.05 are shown in bold.

## Discussion

It was striking that the changes in schizophrenia were often quite different, even opposite, for individual miRNAs measured in whole tissue (Experiment 1) vs. synaptosomes (Experiment 3) of the same brain samples. For example, mir-219-3p and mir-219-5p were up-regulated in whole tissue (48% and 70% increase, respectively) (Table 2) but both were strongly down-regulated in synaptosomes (by 44% (p = 0.013) and 64% (p = 0.0098), respectively). Note that mir-219 is expressed in both neurons and oligodendrocytes [35] while mir-219-5p (but not mir-219-3p) is highly enriched in synaptosomes. This underscores the point that disease-related miRNA changes observed in whole tissue derive from a mixture of cell types (and indeed, may derive from a change in the cell types present, e.g. due to cell shrinkage, gliosis or invasion by microglia), whereas synaptosomes are more selectively informative regarding neurons and the synaptic compartment.

The down-regulation of mir-219 just mentioned is also intriguing because this miRNA is highly enriched in brain relative to other tissues, and mir-219-5p is the most highly enriched miRNA in synaptic fractions (5-fold), while at the same time it is the most strongly down-regulated miRNA in schizophrenia synaptosomes (70% decrease). It has been reported that dizocilpine, a highly selective phencyclidine-like NMDA-R antagonist that can rapidly produce schizophrenia-like behavioral deficits in mice, causes a severe down-regulation of mir-219 in frontal cortex of mice, an effect reversed by antipsychotic agents [36]. Furthermore, mir-219 targets CaMKIIγ, a member of the NMDA receptor signaling pathway, and mice treated with LNA-anti-miR-219 displayed markedly worsened dizocilpine-induced hyperlocomotion and stereotypy, suggesting that mir-219 down-regulation observed in postmortem PFC is functionally relevant to schizophrenia-like phenotypes [36].

## Summary and Conclusions

1. In this paper, we characterized the expression of 377 miRNAs in human PFC obtained from the Stanley Neuropathology Consortium, using high-throughput RT-PCR (TLDA) plates. The changes in expressed miRNA were partially shared between schizophrenia and bipolar disorder, and between bipolar disorder and depression. This agrees with other studies (including other sequencing and gene expression studies) that have documented shared risk determinants and phenotypic abnormalities (e.g., [37]). A relatively small number (2 to 20) of miRNAs showed significant changes in schizophrenia, bipolar disorder and major depression when tested individually by t-test and non-parametric Mann-Whitney U test. In schizophrenia, a subset of up-regulated miRNAs (mir-17-5p, 18a, 106a, 106b, and 590-5p) shared 5′- and 3′-seed sequences, suggesting some coordination in their actions. Some of the up-regulated miRNAs were reported to be expressed predominantly in white matter, whereas down-regulated miRNAs tended to be those which are synaptically enriched.
2. Deep sequencing gives a substantially different picture of miRNA expression than is provided by TLDA plates. In more than 20% of cases, the most abundant miRNA sequence observed in PFC by deep sequencing is NOT the canonical sequence listed in miRBase, but is (usually) a 3′-variant. This is crucial to document since some popular systems, e.g. TaqMan, depend critically on knowing the exact 3′-end of the miRNA. In about 5% of cases, the most abundant sequence is a 5′-variant that exhibits a shifted 5′-seed relative to the canonical miRBase entry. This is crucial to document since target predictions depend critically on knowing position 2–8 of the miRNA.
3. Purified synaptosomes give a unique probe into the biology of the synaptic compartment in human brain. Like synaptosomes prepared from fresh mouse brain, human synaptosomes showed enrichment of a subset of miRNAs relative to whole PFC tissue. Deep sequencing of synaptosomes revealed that there is a selective down-regulation of miRNAs in schizophrenia, that disproportionately affects synaptically enriched miRNAs. These findings point to some deficit in miRNA biogenesis, transport, processing or turnover that is selective for the synaptic compartment. Individual miRNAs often gave divergent, even opposite changes in schizophrenia when measured in whole PFC vs. purified synaptosomes.
4. Besides miRNAs, several other small RNAs showed significant changes in schizophrenia synaptosomes. In particular, a novel class of small RNAs derived from snoRNAs and other ncRNAs had been described in mouse, that are strongly induced during learning [25], [32]. We now find that this class of RNAs is also expressed in man, and at least one member was significantly down-regulated in schizophrenia.

The present report needs replication in additional cohorts and brain regions. In the future, it will be interesting to examine the mechanisms by which synaptic miRNAs are dysregulated, to test whether they contribute actively to pathogenesis, and to learn whether they can be modeled in animal studies.

## Supporting Information

File S1 TLDA expression values (Experiment 1). Sheet 1: Median-normalized Ct values of expressed miRNAs, removing outlier values and samples. Sheet 2: raw Ct values.
(XLSX)

File S2 Deep sequencing of human miRNAs (Experiment 2∶3 male and 3 female PFC and synaptosomes prepared from a pool of the 3 males).
(XLSX)

File S3 Deep sequencing of synaptosome small RNAs in controls vs. schizophrenia. Sheet 1: Number of total and mappable reads in each sample. Sheet 2: Reads aligning uniquely to the human genome, 15–40 nt., within an annotated RefGene locus, filtered to include only sequences expressed >10 reads in at least one sample. Shows both raw and normalized counts.
(XLSX)

File S4 Deep sequencing of synaptosome small RNAs in controls vs. schizophrenia. Reads aligning to multiple (2–20) loci.
(XLSX)

File S5 Supporting information for the Western blots used to create figure 2A. Human synaptosomes were blotted using antibodies against PSD-95, synapsin I and PCNA; the photos are shown without any cropping or image manipulation, superimposed over the blots themselves containing visible MW markers. The molecular weight markers (Bio-Rad dual color 161–0374) from top to bottom are: 250, 150, 100, 75(red), 50, 37, 25 (red), 20, 15, and 10 kDa. Arrowheads indicate the position of the protein of interest on each blot. As in figure 2A, lanes within each blot are: left, total homogenate; center, 20,000×20 min supernatant; right, synaptosomes.
(TIF)
