# Supplementary figures and images for: Expression of microRNAs and Other Small RNAs in Prefrontal Cortex in Schizophrenia, Bipolar Disorder and Depressed Subjects
Source: PLoS One. 2014 Jan 27;9(1):e86469. doi: 10.1371/journal.pone.0086469 (PMC3903529; doi:10.1371/journal.pone.0086469)

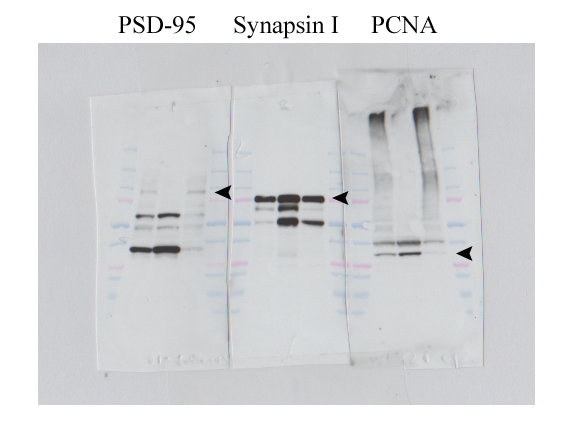

Supplement: File S5 — Supporting information for the Western blots used to create figure 2A. Human synaptosomes were blotted using antibodies against PSD-95, synapsin I and PCNA; the photos are shown without any cropping or image manipulation, superimposed over the blots themselves containing visible MW markers. The molecular weight markers (Bio-Rad dual color 161–0374) from top to bottom are: 250, 150, 100, 75(red), 50, 37, 25 (red), 20, 15, and 10 kDa. Arrowheads indicate the position of the protein of interest on each blot. As in figure 2A, lanes within each blot are: left, total homogenate; center, 20,000×20 min supernatant; right, synaptosomes. (TIF) [file pone.0086469.s005.tif]
